# Inside the nation’s largest mental health institution: a prevalence study in a state prison system

**DOI:** 10.1186/s12889-017-4257-0

**Published:** 2017-04-20

**Authors:** Tala Al-Rousan, Linda Rubenstein, Bruce Sieleni, Harbans Deol, Robert B. Wallace

**Affiliations:** 1 Department of Global Health, Harvard T.H. Chan School of Public Health, 665 Huntington Avenue, Building 1, Room 1107, Boston, MA 02115 USA; 2 Department of Epidemiology, University of Iowa School of Public Health, Iowa City, IA USA; 3 Department of Corrections, Iowa Medical and Classification Center, Coralville, IA USA; 4 Department of Psychiatry, University of Iowa Carver College of Medicine, Iowa City, IA USA

**Keywords:** Aging, Prisoners, Inmates, Correctional, Mental health

## Abstract

**Background:**

The United States has the highest incarceration rate in the world which has created a public health crisis. Correctional facilities have become a front line for mental health care. Public health research in this setting could inform criminal justice reform.

We determined prevalence rates for mental illnesses and related comorbidities among all inmates in a state prison system.

**Methods:**

Cross-sectional study using the Iowa Corrections Offender Network which contains health records of all inmates in Iowa. The point prevalence of both ICD-9 and DSM-IV codes for mental illnesses, timing of diagnosis and interval between incarceration and mental illness diagnosis were determined.

**Results:**

The average inmate (*N* = 8574) age was 36.7 ± 12.4 years; 17% were ≥50 years. The majority of inmates were men (91%) and white (65%).Obesity was prevalent in 38% of inmates, and 51% had a history of smoking. Almost half of inmates were diagnosed with a mental illness (48%), of whom, 29% had a serious mental illness (41% of all females and 27% of all males), and 26% had a history of a substance use disorder. Females had higher odds of having both a mental illness and substance use disorder. Almost all mental illness diagnoses were first made during incarceration (99%). The mean interval to diagnosis of depression, anxiety, PTSD and personality disorders were 26, 24, 21 and 29 months respectively. Almost 90% of mental illnesses were recognized by the 6^th^ year of incarceration. The mean interval from incarceration to first diagnosis (recognition) of a substance abuse history was 11 months.

**Conclusions:**

There is a substantial burden of mental illness among inmates. Racial, age and gender disparities in mental health care are coupled with a general delay in diagnosis and treatment. A large part of understanding the mental health problem in this country starts at prisons.

**Electronic supplementary material:**

The online version of this article (doi:10.1186/s12889-017-4257-0) contains supplementary material, which is available to authorized users.

## Background

Over twenty million Americans are currently or have been incarcerated, the highest rate in the world. In the US in 2013, there were almost 2.3 million people incarcerated in prisons and jails, one in every 110 adults [1]. The mentally ill are overrepresented in correctional settings at estimated rates ranging from two to four times the general population [2]. As result, there are now ten times more individuals with Serious Mental Illnesses (SMI) in prisons and jails than there are in state mental hospitals [3]. Incarceration of people with mental illness is a major public health issue, with social, clinical and economic implications. The balance between public safety and human rights has left corrections services with challenges in providing appropriate care for these patients.

Previous research in this field, such as examining the relationship between the numbers of psychiatric hospital beds in relation to number of inmates [4] and describing recidivism of the mentally ill after release, [5–8] has improved our understanding of the mental health burden in prisons, but a more detailed look is needed. For example, little is known about actual prevalence rates during incarceration across different age, gender and ethnic groups, or the association between substance abuse and other mental illnesses, and general medical co-morbidity.

This study is one a few that used statewide electronic correctional health records to better understand the occurrence of mental illness among prisoners. We also explored the interval between dates of incarceration and psychiatric diagnosis and suggested reasons behind disparities in care and ways to improve it. To our knowledge, only a few studies have investigated this issue [9]. We explored the differences in age distribution in regards to mental illnesses since older prisoners are the fastest growing subgroup with the largest health expenditures within correctional systems [10, 11]. The association with substance use history and the distribution of these illnesses among different demographic subgroups was also described [12–14]. The aims of this study were to illustrate the burden of mental illness in a state prison using the prison’s own data system, profile mental illness and related comorbidities across different subgroups, and determine the vulnerability of these subgroups suggesting ways to help deal with this public health encumbrance.

## Methods

### Study population

The Iowa Department of Corrections (IDOC) collects health and other data on all inmates upon admission to the state prison system during processing at a single site, the Iowa Medical and Classification Center. Using data retrieved from the Iowa Corrections Offender Network, which is the electronic offender management system for staff across the entire statewide corrections system, we obtained and analyzed cross-sectional prevalence data on all IDOC inmates. The data file contained health information as of February 17, 2015, and that date was used as the point prevalence for analysis. The extracted, anonymized data files contained demographic and other characteristics as well as the International Classification of Diseases, 9^th^ Revision (ICD-9) codes for all diagnoses. A separate file contained the Diagnostic and Statistical Manual of Mental Disorders (DSM-IV) codes. The final sample size for all subjects in this study was 8574. The University of Iowa Institutional Review Board approved this study.

## Study variables

Demographic variables included race/ethnicity, gender, birth year, marital status and educational attainment. In this analysis, older inmates were those ≥50 years. Body Mass Index (BMI) was calculated with the formula weight in kilograms divided by the square of height in meters. History of tobacco use (Yes/No), pack-years of cigarette exposure (calculated by multiplying the number of packs of cigarettes smoked per day by the number of years the inmate smoked) and mean of number of years using any smokeless tobacco products reflect data from the initial screening upon admission to the IDOC. The number of general medical conditions was classified into: none, one, two-three and four or more. History of any substance use disorder was diagnosed by the Iowa Medical and Classification Center staff. General medical conditions were determined using ICD-9-CM codes [15]. For this study, medical conditions were classified using three-digit codes, subsuming all four and five digit codes.

With regard to incarceration and crime classifications, supervision status was classified into: prison or work release. Work release is defined by the IDOC as “granting inmates sentenced to an institution under its jurisdiction the privilege of leaving actual confinement during necessary and reasonable hours for the purpose of working at gainful employment” within the prison facility. Crime classification reflected the maximum penalties and included: Life in prison (A Felony), 25–50 years in prison (B Felony), 10 years (C Felony), 5 years (D Felony), 2 years (aggravated misdemeanor) and other felonies (variable penalties that range between 1 and 2 years). Sentence in years reflected length of stay and was determined by Most Serious Conviction Category. Type of crime was classified by the most common crimes, which included: drug, violent, public order, property or other. Commitment indicator included: first, second or more than two terms. Mental illnesses were presented based on DSM-IV codes [16] and ICD-9 codes for each inmate. Recently, the IDOC switched officially from using DSM-IV to using the ICD-9 coding system for psychiatric illnesses. The DSM-IV has a cross reference to ICD-9 codes [17]. Some subjects had an ICD-9 code for mental illness but not a DSM-IV code, so we also reported the prevalence of mental illnesses using information from both DSM-IV and ICD-9 codes, and all diagnoses were manually reviewed to avoid any duplicate counting. Mental illness status was classified into: current, remission and resolved. Date of admission to prison and date of mental illness diagnosis were provided in individual records. The difference between the two dates yielded the interval between current incarceration and diagnosis. The numbers and odds ratios for inmates with a history of substance use also having mental illness were reported.

### Statistical analysis

Descriptive statistics were generated using frequencies and percents for categorical variables and means and standard deviations (SD) for continuous variables. The *p* values in Table 1 were unadjusted, while *p*-values in Tables 2 and 4 were adjusted for age group and gender using Cochran-Mantel-Haenszel methods. Two-sided tests with a *p* value ≤0.05 were considered statistically significant. All analyses were performed using SAS version 9.3 (SAS Institute Inc., Cary, North Carolina).

**Table 1:** Demographic characteristics of the prison’s population (*n* = 8574)

Characteristics	Younger *n* = 7107	Older *n* = 1467	All *n* = 8574	*p* value^a^ Younger vs older
Age, mean (SD) in years	32.4 (8.4)	57.2 (6.8)	36.7 (12.4)	
Age, range in years	16–49	50–88	16–88	
Gender				0.003
Female	637 (9.0)	97 (6.6)	734 (8.6)	
Male	6470 (91.0)	1370 (93.4)	7840 (91.4)	
Race				<0.0001
Black	1913 (26.9)	309 (21.1)	2222 (25.9)	
Hispanic	507 (7.1)	45 (3.1)	552 (6.4)	
White	4473 (62.9)	1089 (74.2)	5562 (64.9)	
Other	214 (3.0)	24 (1.6)	238 (2.8)	
Marital status				<0.0001
Married	1199 (17.0)	408 (28.1)	1607 (18.9)	
Single	953 (13.5)	619 (42.6)	1572 (18.5)	
Divorced or widowed	4884 (69.4)	425 (29.3)	5309 (62.6)	
Highest level of education				<0.0001
College graduate	57 (0.8)	43 (3.0)	100 (1.2)	
Some college	142 (2.1)	66 (4.6)	208 (2.5)	
High school/ equivalent	5266 (76.0)	991 (69.5)	6257 (74.9)	
Less than high school	1300 (18.8)	270 (18.9)	1570 (18.8)	
Other	160 (2.3)	56 (3.9)	216 (2.6)	
Body mass index, kg/m^2^				<0.0001
< 18.5 (underweight)	18 (0.2)	4 (0.3)	22 (0.3)	
18.5- < 25.0 (normal)	1697 (23.9)	191 (13.0)	1888 (22.0)	
25- < 30 (overweight)	2848 (40.1)	594 (40.5)	3442 (40.1)	
≥ 30 (obese)	2544 (35.8)	678 (46.2)	3222 (37.6)	
History of tobacco use	3864 (54.4)	562 (38.1)	4426 (51.6)	<0.0001
Pack-years for smokers only, mean (SD)	13.3 (11.2)	34.9 (23.3)	16.0 (15.2)	<0.0001
Smokeless years, mean (SD)	6.6 (6.9)	18.4 (16.1)	7.8 (9.0)	<0.0001
Medical Conditions, mean (SD)	1.1 (1.3)	2.6 (2.1)	1.4 (1.6)	<0.0001
Chronic Medical Conditions				<0.0001
0	2924 (41.4)	268 (18.3)	3192 (37.2)	
1	2286 (32.7)	297(20.3)	2583 (30.1)	
2–3	1500 (21.1)	465 (31.7)	1965 (22.9)	
≥ 4	397 (5.6)	437 (29.8)	834 (9.7)	

^a^ *p*-values are from Fisher’s exact test or the Pearson chi-square statistic

Values represent numbers and percentages unless indicated otherwise

**Table 2:** History of mental illness in younger and older prisoners (*n* = 8574)

Mental disorders from the ICD9 and DSM	Younger *n* = 7107	Older *n* = 1467	All *n* = 8574	OR (95% CI)^a^ Reference = older
Mental illness (DSM and ICD9)	3448 (48.5)	645 (44.0)	4093 (47.7)	1.3 (1.2–1.5)
Mental illness (DSM)	3222 (45.3)	570 (38.9)	3792 (44.2)	1.4 (1.3–1.6)
Mental illness (ICD9 only)	226 (3.2)	75 (5.1)	301 (3.5)	0.7 (0.5–0.9)
Serious mental illness^b^ (DSM and ICD9)	2048 (28.8)	404 (27.5)	2452 (28.6)	1.1 (0.99–1.3)
Serious mental illness (DSM)	1976 (27.8)	386 (26.3)	2362 (27.5)	1.1 (1.0–1.3)
Serious mental illness (ICD9 only)	72 (1.0)	18 (1.2)	90 (1.0)	ne
Substance use^c^ (DSM and ICD9)	1928 (27.1)	312 (21.3)	2240 (26.1)	1.5 (1.3–1.7)
Substance use (DSM)	1791 (25.2)	286 (19.5)	2077 (24.2)	1.5 (1.3–1.7)
Substance use (ICD9 only)	137(1.9)	26 (1.8)	163 (1.9)	1.2 (0.8–1.8)
Depression, major depressive disorders	1267 (17.8)	294 (20.0)	1561 (18.2)	0.9 (0.8–1.1)
Anxiety, general anxiety, panic disorders	1222 (17.2)	193 (13.2)	1415 (16.5)	1.5 (1.3–1.8)
Personality disorders	824 (11.6)	135 (9.2)	959 (11.2)	1.3 (1.1–1.6)
Psychosis, psychotic disorders	664 (9.3)	97 (6.6)	761 (8.9)	1.5 (1.2–1.9)
Developmental disabilities	677 (9.5)	45 (3.1)	722 (8.4)	3.5 (2.6–4.8)
Bipolar	579 (8.2)	65 (4.4)	644 (7.5)	2.1 (1.6–2.7)
Post-Traumatic Stress Disorder	459 (6.5)	79 (5.4)	538 (6.3)	1.2 (0.9–1.5)
Schizophrenia	177 (2.5)	77 (5.3)	254 (3.0)	0.5 (0.4–0.6)
Impulse control disorders	160 (2.3)	10 (0.7)	170 (2.0)	3.6 (1.9–6.9)
Dysthymia, neurotic depression	132 (1.9)	45 (3.1)	177 (2.1)	ne
Dementia	29 (0.4)	24 (1.6)	53 (0.6)	ne
Sleep, movement and eating disorders	24 (0.3)	2 (0.1)	26 (0.3)	ne
Sexual disorders, paraphelias	11 (0.2)	3 (0.2)	14 (0.2)	ne
Pervasive developmental disorders	7 (0.1)	0 (0.0)	7 (0.1)	ne
Somatization disorders	0 (0.0)	1 (0.1)	1 (0.0)	ne

*DSM* The Diagnostic and Statistical Manual of Mental Disorders, *ICD9* The International Classification of Diseases, 9th Revision, *ne* not estimable, sample size too small. *CI* confidence interval

^a^Odds Ratios and 95% CI are from logistic regression models adjusting for sex and race/ethnicity. If the (95% CI) includes 1.0, the odds ratio is not statistically significant

^b^Serious mental illness includes bipolar disorders, dementia/organic disorders, depression and major depressive disorders, dysthymia/neurotic depression, psychosis/psychotic disorders, schizophrenia, and substance use disorders

^c^Substance use includes alcohol-induced persisting amnestic disorder, cannabis-induced psychotic disorder, with hallucinations, other (or unknown) substance-induced psychotic disorder with hallucinations, phencyclidine-induced psychotic disorder, with hallucinations, psychotic disorder NOS, substance-induced, alcohol dependence, opioid dependence, sedative/hypnotic/anxiolytic dependence, cocaine dependence, cannabis dependence, amphetamine dependence, other polysubstance abuse, methamphetamine dependence, hallucinogen dependence, inhalant dependence, polysubstance dependence, other (or unknown) dependence, phencyclidine dependence

## Results

Table 1 shows the characteristics of the study population, based on the point prevalence date, for both younger and older inmates. The average inmate age was 36.7 (SD = 12.4) years and the majority were males (91.4%) and white (64.9%). Older inmates (≥ 50 years), comprised 17% of the total cohort. A large proportion of inmates were either overweight (40.1%) or obese (37.6%). More than half of the inmates reported a history of smoking (51.2%) averaging 15.1 pack-years. Younger smokers were more likely to have smoked compared to older inmates (54% vs 38.1%). Approximately 63% had at least one chronic medical condition, and among the older inmates, approximately 30% had four or more, compared to 6% for younger inmates. The distribution of medical conditions among the inmate population are shown in Additional file 1: Table S1. Coronary artery disease was the most prevalent chronic medical condition (15%), particularly among the older inmates, followed by hypertension (13%), hyperlipidemia (9%) and hepatitis C (8.3%).

Additional file 1: Table S2 shows the crime and incarceration status of the inmate population. The mean and median sentences were 24.0 and 12.0 years respectively. Nearly half of inmates were convicted for violent crimes (47.8%), with the next most common being drug-related offenses (22.6%). Older inmates had a higher proportion of violent crimes and a somewhat lower proportion of offenses related to drugs. Only a small minority had work release status (6.1%).

Table 2 shows the distribution of mental illness diagnoses according to age group. The odds ratios reflect the relative prevalence among the younger versus the older age groups, adjusted for sex and race/ethnicity. Overall, almost half of the inmates had a history or a diagnosis of one or more mental illnesses (48%). Of female inmates, 60% had a mental illness diagnosis, compared to 46.6% of males (data not shown). Almost a third of all inmates had a Serious Mental Illness (SMI) (29%) (Table 2) and a similar proportion had history of substance abuse (26%). SMIs were relatively more prevalent in females (41%) than males (27%).

For specific mental illnesses, depression and major depressive disorders were the most prevalent conditions, present in 18% of all inmates and accounting for 38% of all the mentally ill inmates (Table 2). The next most common were anxiety and panic disorders, present in 17% of inmates. With regard to sex-specific illnesses (data not shown) females were more likely to be diagnosed with substance abuse, depression and major depressive disorders, developmental disabilities, bipolar disorder, PTSD and sleep, movement and eating disorders. Males were more likely to be diagnosed with impulse control disorders and dysthymia or neurotic depression. Younger inmates were more likely to be diagnosed with substance abuse, anxiety and panic disorders, personality disorders, psychotic disorders, developmental disabilities, bipolar disorder, dysthymia and neurotic depression and impulse control disorders.

**Fig. 1:**
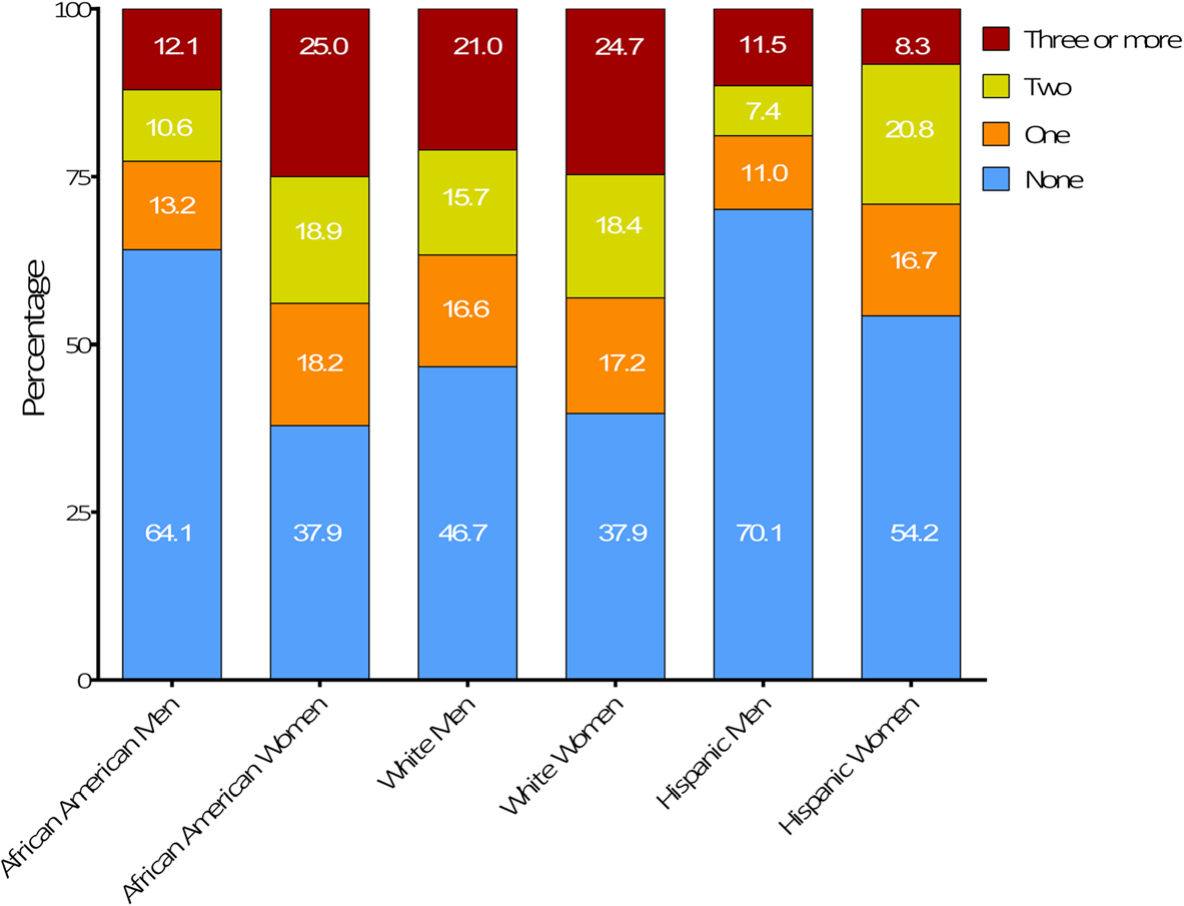
All prevalence rates are cross-sectional. There were 2090 African American men, 64.1% of which has no mental illness. Women in general exhibited the highest burden of mental illness compared to other racial groups. Quarter of the female African American inmates had three or more mental illness diagnoses. These prevalence rates were similar to white women (*n* = 554) of which 24.7% had three or more mental illnesses. More than half of the white men (*n* = 5008) had a mental illness diagnosis which is the highest number compared to rates in other racial groups. Percentage of inmates diagnosed with mental illness in Iowa by race and gender

Figure 1 shows the distribution of mental illnesses counts across race/ethnicity and gender groups. Among the three major race/ethnicity groups, females were more likely to have one or more mental illnesses, but among females, Hispanics had a lower proportion with such illnesses. Among males, whites were more likely to have one or more mental conditions than African-Americans or Hispanics.

Table 3 displays the prevalence of a substance abuse history and odds ratios for those rates, both crude and adjusted for inmate demographic characteristics. Overall, nearly half of those with a mental illness also had a history of substance abuse (48.5%). Having any of the mental conditions was associated with an eight-fold increased odds of also having a substance abuse history. Higher odds ratios were also seen associated with SMIs, anxiety and panic disorders and major depressive illnesses. For all mental conditions where the odds ratios of associated substance abuse were calculable, odds of substance abuse were at least 2.5.

**Table 3:** Odds ratios (95% CI) for the association of history of substance use disorder^a^ with mental illness

Mental disorders	Substance users with disorder, n (%)	Odds Ratio (95% CI)^b^	
		Unadjusted	Adjusted
Any mental disorder (excluding substance abuse)	1747 (48.5)	8.5 (7.6–9.6)	8.1 (7.2–9.1)
Serious mental disorder^c^	1198 (53.5)	4.7 (4.2–5.2)	4.4 (4.0–4.9)
Anxiety, general anxiety, panic disorders	766 (34.2)	4.6 (4.0–5.1)	4.2 (3.7–5-4.8)
Depression, major depressive disorders	711 (31.7)	3.0 (2.7–3.4)	2.8 (2.5–3.2)
Personality disorders	520 (23.2)	4.1 (3.5–4.7)	4.0 (3.5–4.6)
Psychosis, psychotic disorders	406 (18.1)	3.7 (3.2–4.3)	3.8 (3.2–4.4)
Developmental disabilities	360 (16.1)	3.2 (2.7–3.7)	2.9 (2.5–3.4)
Bipolar	358 (16.0)	4.0 (3.4–4.7)	3.6 (3.1–4.3)
Post-Traumatic Stress Disorder	254 (11.3)	2.7 (2.3–3.3)	2.6 (2.2–3.1)
Schizophrenia	117 (5.2)	2.5 (1.9–3.2)	2.8 (2.1–3.6)
Impulse control disorders	87 (3.9)	3.0 (2.2–4.1)	2.9 (2.1–4.0)
Dysthymia, neurotic depression	83 (3.7)	2.6 (1.9–3.4)	2.5 (1.8–3.4)
Sleep, movement and eating disorders	12 (0.5)	ne	ne
Sexual disorders, paraphilia	2 (0.1)	ne	ne
Pervasive developmental disorders	2 (0.1)	ne	ne

Values represent numbers and percentages

*ne* not estimable

^a^Substance use disorder as derived from the ICD9/DSM codes and includes alcohol-induced persisting amnestic disorder, cannabis-induced psychotic disorder, with hallucinations, other (or unknown) substance-induced psychotic disorder with hallucinations, phencyclidine-induced psychotic disorder, with hallucinations, psychotic disorder NOS, substance-induced, alcohol dependence, opioid dependence, sedative/hypnotic/anxiolytic dependence, cocaine dependence, cannabis dependence, amphetamine dependence, other polysubstance abuse, methamphetamine dependence, hallucinogen dependence, inhalant dependence, polysubstance dependence, other (or unknown) dependence, phencyclidine dependence;

^b^Odds ratios were generated in logistic regression models adjusted for age, gender, and race/ethnicity; Odds ratios are statistically significant if the 95% CI does not include 1.0

^c^Serious mental illness includes bipolar disorders, dementia/organic disorders, depression and major depressive disorders, dysthymia/neurotic depression, psychosis/psychotic disorders, schizophrenia, and substance use disorders

Additional file 1: Table S3 shows the numbers and rates of mental disorders among younger and older inmates, according to whether the disorders were considered currently active or resolved/in remission. Overall, in both younger and older age groups, the majority of all conditions ever diagnosed were “current,” that is, active. This was true for both males and females (data not shown). Particularly, older inmates were more likely to be diagnosed during incarceration (74%).

Table 4 and Additional file 1: Figure S1 show the diagnoses of all mental illnesses for the inmates’ life duration in prison since incarceration. The mean interval to first diagnosis of substance abuse and bipolar disorders was 11 months since admission. The mean interval to diagnosis of depression, anxiety, PTSD and personality disorders were 26, 24, 21 and 29 months respectively. For psychosis, the mean duration was 14 months and for developmental disabilities it was 16 months. The longest mean durations to diagnosis were for schizophrenia and dysthymia, 52 and 45 months respectively. The shortest duration was to the diagnosis of dementia (7 months). As shown in Additional file 1: Figure S1, almost 90% of mental illnesses were diagnosed (i.e., appeared in the clinical record) by the 6^th^ year of incarceration.

**Table 4:** Duration in months to the first documentation of mental illness made after prison entry^a^

Mental disorders	First diagnosis^b^ *n* = 4044	Months		Percent diagnosed in months			
	Frequency (%)	Mean (SD)	Median [range]	≤1	>1–6	> 6–24	> 24
Substance abuse^c^	1130 (27.9)	10.3 (36.6)	0.5 [0.25–402]	72.2	13.5	6.0	8.3
Depression	729 (18.0)	26.2 (65.0)	0.75 [0.25–483]	57.2	14.9	8.0	19.9
Anxiety	616 (15.2)	24.4 (57.6)	1.0 [0.25–385]	52.1	18.3	12.0	17.5
Personality disorders	366 (9.1)	29.5 (59.0)	3.0 [0.25–354]	39.1	21.3	13.9	25.7
Psychosis and psychotic disorders	280 (6.9)	13.7 (36.3)	0.75 [0.25–290]	60.7	16.4	10.0	12.9
Developmental disabilities	250 (6.2)	15.5 (39.4)	2.0 [0.25–268]	42.4	28.4	15.6	13.6
Post-traumatic stress disorders	247 (6.1)	21.0 (49.7)	1.0 [0.25–322]	53.0	19.8	7.3	19.8
Bipolar	175 (4.3)	11.4 (24.1)	1.0 [0.25–143]	52.0	17.7	18.3	12.0
Schizophrenia	91 (2.3)	52.2 (90.2	5.0 [0.25–354]	44.0	6.6	11.0	38.5
Dysthymia/neurotic depression	66 (1.6)	44.7 (57.7)	18.0 [0.25–216]	31.8	12.1	10.6	45.5
Impulse control	55 (1.4)	18.6 (26.7)	5.0 [0.25–100]	36.4	16.4	23.6	23.6
Dementia	18 (0.5)	6.7 (8.1))	3.5 [0.25–30]	33.3	33.3	27.8	5.6
Sexual disorders, paraphilia	9 (0.2)	23.9 (51.2)	1.0 [0.25–158]	55.6	0	22.2	22.2
Sleep, movement and eating disorders	9 (0.2)	41.1 (53.1)	4.0 [0.25–144]	33.3	22.2	0	44.4
Pervasive developmental disorders	3 (0.1)	0.4 (0.3)	0.25 [0.25–0.75]	100.0	0	0	0

*SD* standard deviation

^a^excluding any prisoners with mental illness diagnosed before incarceration

^b^The first documentation of mental illness included 1 to 5 different types of mental disorders; the denominator for the total frequency may include multiple mental disorders per prisoner

^c^Substance use includes alcohol-induced persisting amnestic disorder, cannabis-induced psychotic disorder, with hallucinations, other (or unknown) substance-induced psychotic disorder with hallucinations, phencyclidine-induced psychotic disorder, with hallucinations, psychotic disorder NOS, substance-induced, alcohol dependence, opioid dependence, sedative/hypnotic/anxiolytic dependence, cocaine dependence, cannabis dependence, amphetamine dependence, other polysubstance abuse, methamphetamine dependence, hallucinogen dependence, inhalant dependence, polysubstance dependence, other (or unknown) dependence, phencyclidine dependence

## Discussion

Mental health conditions constitute a substantial burden among inmates in correctional institutions, and this issue has gained increasing attention in recent years. Within the state penitentiaries in Iowa, we found the prevalence rate for mental illnesses to be nearly 50%, similar to prevalence rates reported by other states [18]. However, US national estimates mental illness rates are somewhat dated, as the most recent survey by the Bureau of Justice Statistics (BJS) was performed in 2004 for federal inmates and in 2002 for jail inmates [18].

While only representing the experience of one state, this study provides recent and representative estimates for the prevalence of mental health problems among individuals involved in the criminal justice system. In previous studies within federal prisons and jails, inmates have been asked to complete a modified clinical interview based on the DSM-IV [19–21]. Inmates in mental hospitals or otherwise physically or mentally unable to complete the surveys were excluded from such studies. Despite this, it has been estimated in the BJS study that 56% of state inmates had a mental health problem, similar to our own findings despite differing methods. Also, we found that illness rates were higher among female than males, consistent with BJS reports. In this study, the presence of mental illness was associated with an eight-fold increased odds of a substance use and abuse history.

In this cross-sectional prevalence study, those with diagnosed mental disorders were much more likely to be under active treatment than designated as resolved or in remission. There is evidence that inmates with a mental condition are more likely to have been charged with breaking correctional facility rules [18]. Also, they are more likely to be injured in a fight and be charged with a physical or verbal assault on correctional staff or another inmate. These behaviors may promote more concurrent mental illness diagnoses in the clinical records, in part, because these inmates receive more clinical attention. Thus, it is not surprising that these illnesses are generally very costly to treat, and patients are at great risk for recidivism, hospitalization, and suicide upon release [22]. These experiences have been previously described, as well as the wave of mass incarceration and increasing deinstitutionalization of the mentally ill in the community, resulting in an increased prevalence of mental illness in prisons. It is not surprising that mental illness is the leading cause of clinical expenditures in corrections facilities [23].

High rates of mental illnesses among inmates in this study, as well as in other national studies, are likely to be in part because of the reservoir of mental conditions and the inadequate treatment access in the community prior to incarceration [24]. Another possibility is the higher incarceration rates for substance abuse, part of the “War on Drugs” [25]. Substance use and abuse are associated with other psychiatric co-morbidity [26]. Delayed psychiatric diagnosis in many inmates is analogous to findings from other studies [24]. This may be attributed to the following explanations: high rates of undiagnosed conditions at entry, the incomplete transmission of relevant clinical records upon incarceration and highly stressed diagnostic and management resources within the corrections system. It is also possible that some inmates choose to withhold their psychiatric histories because of stigma and other reasons. Another hypothesis is that the prison environment itself may be stressful enough to ignite subclinical mental illnesses during the course of incarceration related to social isolation, violence, lack of support and others [27, 28]. There is an important need for longitudinal research to evaluate and better understand our findings and those of others. Despite potential clinical policy and resource problems within prisons and jails, contrasts with community care dynamics would seem to also be of value.

Black and Hispanic inmates were overrepresented relative to Iowa’s general population which is comparable to other demographic data on incarcerated populations [29]. According to the US Census Bureau, 3.3% of Iowa’s population is African-American [30], in contrast to 26% in the state prison system. Mental health challenges, substance use disorders, and HIV/AIDS disproportionately affect African-Americans in correctional settings [31], consistent with our findings. Minority inmates had mental illness rates generally similar to Whites, but Whites were more likely to have more than one mental illness diagnosis. Of interest, female Hispanic inmates were more likely to have three or more mental conditions in contrast to their White and Black counterparts. Women inmates, especially younger ones, were more likely than men to be diagnosed with a larger variety of mental disorders in this analysis. Women in general were at greater risk than men to be diagnosed with mental illness during incarceration.

Older inmates were less likely to have reported substance abuse and tobacco use than younger inmates. Although, this may reflect the reasons for incarceration, the IDOC corrections staff believe that this may be explained in part by inadequacies of the IDOC record system before transition to electronic health records. However, to improve preventive care in general, it may be important to record inmate smoking and drug use at more frequent intervals. Differences in smoking and other substance use history between older and younger inmates may also be due to differences in the types and prevalence of mental conditions in these two groups, as shown in this study, as well as cohort variation.

Substance use disorders are commonly seen in incarcerated populations; in this study a history was recorded in 26.1% of all inmates, and 50% higher in the younger inmate population. Substance use disorders were more common over eight times in the presence of any other mental illness diagnosis [32, 33]. Mortality in people with mental illness is far higher in individuals with substance use disorders [30, 34, 35]. Mortality-reducing interventions should focus on patients with dual diagnosis.

The IDOC electronic medical record system clearly enables more detailed epidemiological studies of inmate health status. However, the results reported here should be interpreted in light of a few potential limitations. There may be variation in the accuracy of disease diagnosis and coding by physicians and other staff, but it is not possible to test for this in the current situation. A companion issue in assessing completeness of psychiatric and other diagnoses is the problem of access to clinical records from community sources. A related potential limitation in diagnostic accuracy is that prevalence rates of mental illness may be underestimated since physicians have limited time and resources for clinical interviews and all evaluations may not be equally thorough, exacerbated by frequent transfer of inmates between prisons. Finally, it may at times be difficult to determine the sources of mental illness and other diagnoses, whether from various community clinical services, or new patient assessments or accounts. There may be some inconsistency again because of difficulty in acquiring prior medical records for use in the IDOC. However, even if prior records are acquired, there may be variation in completeness as it has recently been shown that community electronic health records may have missing behavioral health and other data [36].

## Conclusions

There is a high prevalence of mental illness and related comorbidity in the prison population in Iowa. Subpopulations such as women, minorities and older inmates have distinctive patterns of these diseases. Timely diagnosis at intake and awareness of racial and other disparities are critical to improving health care in these patients. Historically, public health and corrections systems have operated with varying priorities. More accurate and timely usage of data may assist in improving this situation.

## Additional file

Additional file 1: Figure S1. This figure is important to estimate the average time since incarceration when a mental illness diagnosis is made. The denominator is the number of mentally ill inmates (*n* = 8574). This is a retrospective look at intervals of time calculating time of mental illness diagnosis minus date of beginning of incarceration. Almost half of all diagnoses of the mentally ill were made around week 3 since incarceration. However, 75% of all mental illness diagnoses took up to 6 months to be made. **Table S1.** History of medical conditions among prison’s population (*N* = 8574). **Table S2.** Incarceration characteristics of the prison’s population (*n* = 8574). **Table S3.** Mental disorder status (current compared to resolved/in remission) ^a^ in younger and older prisoners. **Table S4.** The ICD-9-CM codes used to define chronic medical conditions. (DOCX 40 kb)

## Abbreviations

BMI: Body mass index
CI: Confidence interval
DSM: Diagnostic and statistical manual of mental disorders
ICD: International classification of disease
IDOC: Iowa Department of Corrections
IMCC: Iowa Medical and Classification Center
OR: Odds ratio
SD: Standard deviation
SMI: Serious mental illness
