# Supplementary material for: Inside the nation’s largest mental health institution: a prevalence study in a state prison system
Source: BMC Public Health. 2017 Apr 20;17:342. doi: 10.1186/s12889-017-4257-0 (PMC5397789; doi:10.1186/s12889-017-4257-0)
Supplement: Additional file 1: Figure S1. — This figure is important to estimate the average time since incarceration when a mental illness diagnosis is made. The denominator is the number of mentally ill inmates (n = 8574). This is a retrospective look at intervals of time calculating time of mental illness diagnosis minus date of beginning of incarceration. Almost half of all diagnoses of the mentally ill were made around week 3 since incarceration. However, 75% of all mental illness diagnoses took up to 6 months to be made. Table S1. History of medical conditions among prison’s population (N = 8574). Table S2. Incarceration characteristics of the prison’s population (n = 8574). Table S3. Mental disorder status (current compared to resolved/in remission) a in younger and older prisoners. Table S4. The ICD-9-CM codes used to define chronic medical conditions. (DOCX 40 kb) [file 12889_2017_4257_MOESM1_ESM.docx]

| Table S1. History of medical conditions among prison’s population (N= 8,574) | | | | |
| --- | --- | --- | --- | --- |
| Medical conditions | Younger  n=7,107 | Older  n=1,467 | All  n=8,574 | Younger vs Older p-value^a^ |
| Coronary artery disease | 703 (9.9) | 556 (37.9) | 1259 (14.7) | <0.0001 |
| Hypertension | 618 (8.7) | 470 (32.0) | 1088 (12.7) | <0.0001 |
| Hyperlipidemia | 358 (5.0) | 371 (25.3) | 729 (8.5) | <0.0001 |
| Hepatitis C | 470 (6.6) | 239 (16.3) | 709 (8.3) | <0.0001 |
| Chronic lung disease | 521 (7.3) | 185 (12.6) | 704 (8.2) | <0.0001 |
| Problems with vision | 210 (3.0) | 321 (21.9) | 531 (6.2) | <0.0001 |
| Neurological disease | 312 (4.4) | 91 (6.2) | 403 (4.7) | 0.004 |
| Obesity | 266 (3.7) | 99 (6.7) | 365 (4.3) | <0.0001 |
| Thyroid disease | 143 (2.0) | 101 (6.9) | 244 (2.8) | <0.0001 |
| Back pain | 147 (2.1) | 91 (6.2) | 238 (2.8) | <0.0001 |
| Diabetes type 2 | 161 (2.3) | 172 (11.7) | 333 (3.9) | <0.0001 |
| Arthritis | 76 (1.1) | 119 (8.1) | 195 (2.3) | <0.0001 |
| Tuberculin positive skin test | 89 (1.3) | 17 (1.2) | 106 (1.2) | 0.90 |
| Kidney disease | 30 (0.4) | 51 (3.5) | 81 (0.9) | <0.0001 |
| Sexually transmitted disease | 60 (0.8) | 10 (0.7) | 70 (0.8) | 0.63 |
| Cancer | 32 (0.5) | 37 (2.5) | 69 (0.8) | <0.0001 |
| Problems with hearing | 21 (0.3) | 21 (1.4) | 42 (0.5) | <0.0001 |
| Diabetes mellitus, type 1 | 29 (0.4) | 2 (0.1) | 31 (0.4) | 0.15 |

^a^ p-values from Fisher’s exact test or the Pearson chi-square statistic.

Values represent numbers and percentages.

| Table S2. Incarceration characteristics of the prison’s population (n= 8,574) | | | | |
| --- | --- | --- | --- | --- |
| Characteristics | Younger  n=7,107 | Older  n=1,467 | All  n=8,574 | p-value^a^ Younger vs Older |
| Supervision status |  |  |  | <0.0001 |
| Work release | 474 (6.7) | 51 (3.5) | 525 (6.1) |  |
| Prison | 6633 (93.3) | 1416 (96.5) | 8049 (93.9) |  |
| Crime Classification |  |  |  | <0.0001 |
| A felony | 345 (4.9) | 289 (19.7) | 634 (7.4) |  |
| B felony | 1141 (16.1) | 349 (23.8) | 1490 (17.4) |  |
| C felony | 2434 (34.2) | 302 (20.6) | 2736 (31.9) |  |
| D felony | 1888 (26.6) | 215 (14.7) | 2103 (24.5) |  |
| Aggravated misdemeanor | 579 (8.1) | 85 (5.8) | 664 (7.7) |  |
| Other classes | 720 (10.1) | 227 (15.5) | 947 (11.1) |  |
| Sentence, years, mean (SD) | 20.5 (22.8) | 40.7 (36.8) | 24.0 (26.8) |  |
| Sentence, years |  |  |  | <0.0001 |
| ≤ 7 | 1831 (25.8) | 246 (16.8) | 2077 (24.2) |  |
| >7 to 11 | 1928 (27.1) | 196 (13.4) | 2124 (24.8) |  |
| >11 to 25 | 1990 (28.0) | 363 (24.7) | 2353 (27.4) |  |
| > 25 | 607 (8.5) | 171 (11.7) | 778 (9.1) |  |
| ≥ 50 | 400 (5.6) | 156 (10.6) | 556 (6.5) |  |
| Life sentence | 351 (4.9) | 335 (22.8) | 686 (8.0) |  |
| Type of crime |  |  |  | <0.0001 |
| Drug | 1701 (23.9) | 237 (16.2) | 1938 (22.6) |  |
| Violent | 3156 (44.4) | 939 (64.0) | 4095 (47.8) |  |
| Public order | 486 (6.8) | 113 (7.7) | 599 (7.0) |  |
| Property | 1562 (22.0) | 145 (9.9) | 1707 (19.9) |  |
| Other | 202 (2.8) | 33 (2.2) | 235 (2.7) |  |
| Commitment times |  |  |  | <0.0001 |
| First term | 4792 (67.5) | 817 (55.7) | 5609 (65.5) |  |
| Second term | 1349 (19.0) | 281 (19.1) | 1630 (19.0) |  |
| > Two terms | 957 (13.5) | 369 (25.2) | 1326 (15.5) |  |

^a^ p-values from Fisher’s exact test or the Pearson chi-square statistic.

Values represent numbers and percentages.

| Table S3: Mental disorder status (current compared to resolved/in remission) ^a^ in younger and older prisoners | | | | | |
| --- | --- | --- | --- | --- | --- |
| Mental conditions | Younger | | Older | | OR (95% CI)^b^ reference = older |
|  | Current | Resolved or in remission | Current | Resolved or in remission |  |
| Substance abuse ^c^ | 1168 (61.0 ) | 746 (39.0 ) | 159 (51.5 ) | 150 (48.5 ) | 1.5 (1.2-1.9) |
| Depression, major depressive disorders | 883 (70.0 ) | 379 (30.0 ) | 188 (64.2 ) | 105 (35.8 ) | 1.3 (1.003-1.7) |
| Anxiety, general anxiety, panic disorders | 963 (78.8 ) | 259 (21.2 ) | 147 (76.2 ) | 46 (23.8 ) | 1.1 (0.8-1.6) |
| Personality disorders | 785 (95.3 ) | 39 (4.7 ) | 118 (87.4 ) | 17 (12.6 ) | 3.0 (1.6-5.4) |
| Psychosis, psychotic disorders | 481 (72.9 ) | 179 (27.1 ) | 54 (55.7 ) | 43 (44.3 ) | 2.1 (1.4-3.3) |
| Developmental disabilities | 581 (85.9 ) | 95 (14.1 ) | 38 (84.4 ) | 7 (15.6 ) | 1.2 (0.5-2.8) |
| Bipolar | 479 (83.0 ) | 98 (17.0 ) | 48 (75.0 ) | 16 (25.0 ) | 1.7 (0.9-3.1) |
| Post-Traumatic Stress Disorder | 369 (80.7 ) | 88 (19.3 ) | 54 (68.4 ) | 25 (31.6 ) | 1.9 (1.1-3.2) |
| Schizophrenia | 134 (77.5 ) | 39 (22.5 ) | 59 (78.7 ) | 16 (21.3 ) | 1.0 (0.5-1.9) |
| Impulse control disorders | 122 (76.3 ) | 38 (23.8 ) | 9 (90.0 ) | 1 (10.0 ) | ne |
| Dysthymia, neurotic depression | 66 (50.4 ) | 65 (49.6 ) | 16 (35.6 ) | 29 (64.4 ) | 1.9 (0.9-4.0) |
| Dementia | 21 (72.4 ) | 8 (27.6 ) | 23 (95.8 ) | 1 (4.2 ) | ne |
| Sleep, movement, eating disorders | 15 (62.5 ) | 9 (37.5 ) | 2 (100.0 ) | 0 (0.0 ) | ne |
| Sexual disorders, paraphelias | 10 (90.9 ) | 1 (9.1 ) | 3 (100.0 ) | 0 (0.0 ) | ne |
| Pervasive developmental disorders | 5 (71.4 ) | 2 (28.6 ) | 0 (0.0) | 0 (0.0) | ne |
| ^a^ Totals may differ from other tables due to missing status data;  ^b^ Odds ratios were generated in logistic regression models adjusted for gender and race/ethnicity; Odds ratios are statistically significant if the 95% CI does not include 1.0.  ^c^ Substance use includes alcohol-induced persisting amnestic disorder, cannabis-induced psychotic disorder, with hallucinations, other (or unknown) substance-induced psychotic disorder with hallucinations, phencyclidine-induced psychotic disorder, with hallucinations, psychotic disorder NOS, substance-induced, alcohol dependence, opioid dependence, sedative/hypnotic/anxiolytic dependence, cocaine dependence, cannabis dependence, amphetamine dependence, other polysubstance abuse, methamphetamine dependence, hallucinogen dependence, inhalant dependence, polysubstance dependence, other (or unknown) dependence, phencyclidine dependence;  ne, not estimable.  Values represent numbers and percentages. | | | | | |

Table S4: The ICD-9-CM codes used to define chronic medical conditions:

| Condition | ICD-9-CM codes |
| --- | --- |
| **Cardiovascular disease** | 785.2, 780.2, 785, 785.1, 785.3, 785.9, 786.05, 786.05, 786.59, V43.60, 396.3, 410.9, 411.1, 413.9, 414, 414.9, 416, 421, 424, 424.1, 424.9, 424.99, 425.4, 426.11, 426.7, 427.2, 427.31, 427.89, 427.9, 428, 429.3, 435.9, 436, 437.3, 440.9, 441.9, 443, 443.9, 447.6, V42.7, V44.2, 746.9, 745.4 |
| **Hypertension** | 401.9 |
| **Hepatitis C** | 70.5, 70.51, 99.29 |
| **Hepatitis B** | 70.3 |
| **Hyperlipidemia** | 272.4, 272 |
| **Chronic lung disease** | 486, 490, 492.8, 493.9, 496, 519.3, 519.8 |
| **Chronic kidney disease** | 581.9, 584.9, 585, 592, 593.2, 593.9, 753.12 |
| **Diabetes** | 250, 250.01 |
| **Thyroid disease** | 239.7, 240.9, 241.9, 242, 242.2, 242.9, 244.9, 246. |
| **Substance dependence/ abuse** | 303.9, 304, 304.2, 304.3, 304.4, 304.6, 304.8, 305, 305.1, 305.2, 305.6, 305.7, 305.9, 291.89 |
| **Vision (serious problems with vision only)** | 361- 366.9, 368- 371.6, 377- 379.31 |
| **Hearing** | 386, 388.4, 388.8, 389.1, 389.9 |
| **Sexually Transmitted Diseases (STDs)** | 91.9, 97.9, 795.71, 42, 54.1, 54.43, 54.9, 79.4, 78.19 |
| **Obesity** | 278 |
| **Purified Protein Derivative (PPD+)** | 795.5 |
| **Cancer** | 146.2- 208.9, 232.9, 238.2, 238.4, 239.5, 239.7 |
